# Comparative Analyses of Drilling Force, Temperature, and Damage in Natural and Glass Fiber-Reinforced Al–Epoxy Composites

**DOI:** 10.3390/polym18020229

**Published:** 2026-01-15

**Authors:** Muammer Kına, Uğur Köklü, Sezer Morkavuk, Mustafa Ay, Yalçın Boztoprak, Barkın Bakır, Murat Demiral

**Affiliations:** 1 Department of Mechanical Engineering, Institute of Science, Marmara University, Istanbul 34854, Türkiye; muammer.kina@marmara.edu.tr; 2 Department of Mechanical Engineering, Faculty of Engineering, Karamanoglu Mehmetbey University, Karaman 70100, Türkiye; sezermorkavuk@kmu.edu.tr; 3 Department of Mechanical Engineering, Faculty of Engineering and Architecture, Recep Tayyip Erdogan University, Rize 53100, Türkiye; 4 Department of Mechanical Engineering, Faculty of Technology, Marmara University, Istanbul 34854, Türkiye; muay@marmara.edu.tr (M.A.); barkinbakir@marmara.edu.tr (B.B.); 5 Department of Metallurgy and Materials Engineering, Faculty of Technology, Marmara University, Istanbul 34854, Türkiye; yboztoprak@marmara.edu.tr; 6 College of Engineering and Technology, American University of the Middle East, Egaila 54200, Kuwait; murat.demiral@aum.edu.kw

**Keywords:** natural fiber-reinforced composite, drilling process, temperature measurement

## Abstract

This study examined the drilling performance of five polymer composite systems: three natural fiber (jute, flax, hemp) composites with aluminum particle-reinforced epoxy, one glass fiber-reinforced composite with the same matrix, and an unreinforced aluminum particle-filled epoxy (Al–epoxy). Drilling experiments were performed at spindle speeds of 1500 and 3000 rpm with feed rates of 50, 75, and 100 mm/min in order to evaluate the effect of cutting parameters on the drilling performance. Cutting zone temperatures were measured using thermocouples embedded within the drill bit’s cooling channels, while thrust forces were recorded with a dynamometer. Additionally, hole exit damage and inner hole surface roughness were evaluated to assess machining quality. The results showed that increasing spindle speed reduces thrust forces due to thermal softening of the matrix, whereas natural fiber-reinforced composites generally exhibit higher thrust forces and slightly rougher inner hole surfaces compared to synthetic counterparts. During drilling, the measured thrust forces ranged from 320 to 693 N for the glass fiber-reinforced specimen and from 335 to 702 N for the Al–epoxy specimen, while for natural fiber-reinforced composites the thrust force values were 352–679 N for hemp, 241–719 N for jute, and 571–732 N for flax specimens. Synthetic specimens (glass fiber and Al–epoxy) exhibited comparable cutting temperature ranges (288–371 °C and 248–327 °C, respectively), whereas natural fiber-reinforced composites showed higher and broader temperature ranges of 311–389 °C for hemp, 368–374 °C for jute, and 307–379 °C for flax specimens. The overall results indicated that lower forces were generated during the drilling of synthetic glass fiber-reinforced composites, while among natural fiber-reinforced plastics, flax fiber-reinforced composites stood out by exhibiting a balanced machining response.

## 1. Introduction

Advancements in technology and the growing demands of modern engineering have led to a paradigm shift in materials development, where performance, sustainability, and environmental considerations are equally critical. Traditionally, material selection has focused primarily on mechanical strength, stiffness, and durability. However, contemporary engineering applications increasingly require materials that are lightweight, cost-effective, recyclable, environmentally friendly, and capable of providing high structural performance under diverse operating conditions. Within this context, composite materials—engineered by combining two or more distinct constituents at the macro or micro scale—have emerged as a prominent solution, offering properties that individual materials cannot achieve alone [1,2,3,4]. Their high specific strength, superior fatigue resistance, design flexibility, and adaptability to various manufacturing techniques make composites highly attractive for critical industries such as aerospace, automotive, defense, construction, and renewable energy sectors [1,5].

Fiber-reinforced polymer composites (FRPCs), in particular, have garnered attention due to their exceptional strength-to-weight ratio, corrosion resistance, and ability to tailor mechanical properties through fiber orientation and volume fraction. The use of synthetic fibers, such as glass, carbon, and aramid, has dominated structural applications for decades. Nonetheless, the rising awareness of environmental sustainability and the need to reduce carbon footprint have prompted research into natural fiber-reinforced composites (NFRCs). Fibers derived from plants, such as jute, flax, hemp, sisal, and bamboo, offer significant ecological advantages, including biodegradability, low density, renewability, and reduced energy consumption during production. While NFRCs may exhibit lower absolute mechanical properties compared to traditional synthetic fibers, recent studies have shown that hybridization with high-performance matrices or particulate reinforcements can yield composites with competitive performance [3]. Moreover, the unique lignocellulosic structure of natural fibers introduces complex fiber–matrix interactions that influence mechanical and thermal behavior, making systematic investigation crucial for process optimization and application.

Machining of composites, particularly drilling, is an essential industrial process since many components require fasteners for assembly. Despite its prevalence, drilling FRPCs poses significant challenges due to the heterogeneous, anisotropic, and layered nature of these materials. Unlike metals, composites exhibit low thermal conductivity and low glass transition temperatures, which lead to unique machining-induced defects, including delamination, fiber pull-out, uncut fibers, fiber–matrix debonding, interlaminar cracks, and matrix smearing. These defects can severely compromise structural integrity and dimensional accuracy, emphasizing the need for precise control over machining operations [6,7]. The extent and severity of drilling-induced damage depend heavily on cutting parameters, tool geometry, tool material and coating, and cooling conditions, as well as the specific characteristics of the fiber–matrix system.

During machining processes, the majority of the energy applied to deform the material is converted into heat [8]. In drilling, the cutting temperature at the interface between the drill bit and workpiece critically influences tool wear, hole accuracy, surface finish, and structural integrity of the composite. The generation of heat results from friction, chip formation, fiber cutting, and the viscoelastic response of the polymer matrix. Importantly, fiber type, matrix composition, and fiber–matrix interface characteristics modulate thermal behavior. Cutting speed, feed rate, tool geometry, tool coating, and cooling strategies govern the heat generation and dissipation mechanisms, thereby affecting both drilling performance and damage formation [9,10,11].

Experimental studies on natural fiber-reinforced composites have demonstrated that delamination and other drilling-induced damages are closely tied to machining parameters. For example, low feed rates and moderate spindle speeds have been shown to minimize delamination at both hole entry and exit, while drill point geometry significantly influences thrust force and cutting behavior [3,12,13]. In flax-fiber-reinforced epoxy composites, statistical analyses have identified feed rate as the primary factor affecting delamination, whereas drill diameter exerts the strongest influence on thrust forces. These findings underscore the complex and often competing influences of machining parameters, where optimization for damage minimization may conflict with productivity or tool life [12,13]. Despite these advances, most prior studies have predominantly focused on a limited set of performance metrics—such as thrust force, delamination, or tool wear—without simultaneously addressing the coupled effects of cutting parameters, thermal behavior, and resulting damage.

Accurate measurement of cutting zone temperature is critical for understanding drilling outcomes. Non-contact infrared thermography and embedded or thin-film thermocouples are widely used techniques, providing real-time, spatially resolved data on heat generation and distribution during drilling [14]. These measurements allow researchers to correlate temperature evolution with damage mechanisms, such as matrix softening, fiber pull-out, and delamination propagation. In natural fiber-reinforced composites, additional complexity arises due to the hygroscopic and heterogeneous nature of fibers, which can influence thermal conductivity, fiber–matrix bonding, and localized degradation [15]. Consequently, systematic studies integrating cutting temperature, thrust force, and damage characterization are essential for optimizing machining strategies and ensuring high-quality composite components.

In addition to thrust force and temperature, hole exit quality and inner hole surface morphology are critical indicators of machining performance. Hole exit delamination, often more severe than entry delamination, can compromise assembly integrity and load transfer efficiency. Similarly, inner hole surface roughness affects fastener seating, adhesion, and long-term fatigue behavior. While some studies have addressed hole quality in synthetic-fiber composites, comparative investigations encompassing both natural and synthetic fibers, particularly in combination with particulate-reinforced polymer matrices, are limited. Understanding the interplay between fiber type, cutting parameters, thermal effects, and damage formation is therefore pivotal for both academic research and practical applications.

Although numerous studies have investigated the drilling behavior of natural fiber–reinforced epoxy composites [16,17] and aluminum particle–reinforced composites separately [18], a comprehensive and systematic comparison of these material systems under identical drilling conditions remains limited. The novelty of the present study lies in the direct comparative evaluation of synthetic fiber–reinforced (glass fiber), natural fiber–reinforced (hemp, jute, and flax), and Al–epoxy composites using the same drilling parameters. By simultaneously analyzing thrust force, cutting temperature, and surface roughness, this work provides a unified quantitative assessment of machinability and performance trade-offs among sustainable and conventional composite systems. The results offer new insights into the relative drilling performance of different fiber and particle reinforcements, thereby contributing to material selection and sustainable composite design for machining applications.

## 2. Material and Methods

### 2.1. Materials

In this study, five different composite specimens were produced using flax, hemp, jute, glass fiber, and Al–epoxy. The properties of the composites produced, and the reinforcements used are given in Table 1. As the resin system, a two-component, high-temperature-resistant epoxy commonly used in casting applications—BASAT-A 102520 epoxy and BASAT-B 102520 hardener—was used. The components were mixed at a ratio of 1.3:10, and 44 µm aluminum particles with 98.85% purity were incorporated into the resin at a proportion of 60%. The technical specifications of the epoxy and hardener are provided in Table 2.

Natural fibers typically exhibit high moisture absorption during storage or after processing, which leads to fiber swelling. Swollen fibers, in turn, reduce fiber–matrix adhesion and consequently degrade the mechanical properties of natural fiber-reinforced composites [19]. Therefore, the fibers were cut to dimensions of 250 × 450 mm and dried in an oven at 100 °C for 2 h prior to manufacturing. Prior to composite fabrication, the natural fibers were dried at 100 °C for 2 h to remove free and bound moisture. This temperature–time combination is widely accepted as safe for lignocellulosic fibers and does not induce chemical degradation. For lignocellulosic materials, moisture evaporation constitutes the dominant thermal event within the 30–120 °C range, representing the fundamental goal of the drying stage. Furthermore, hemicellulose degradation typically initiates above 180–220 °C, whereas significant cellulose decomposition is observed in the 240–350 °C range. Although lignin softening may begin at approximately 80 °C, this represents a reversible physical transition rather than permanent chemical degradation. Moisture removal at 100 °C may increase fiber stiffness due to reduced plasticization without compromising structural integrity. Therefore, the drying process is essential and does not adversely affect the fiber structure prior to composite manufacturing.

**Figure 1:**
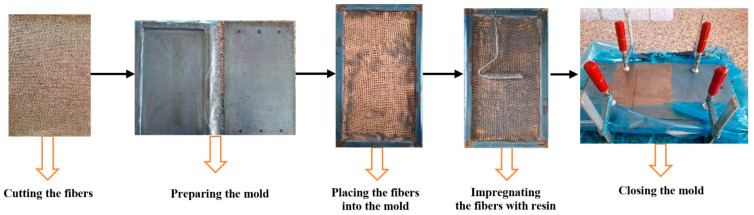
Manufacturing Procedure of the Composite Plates.

The composite specimens were produced using the hand lay-up technique (Figure 1). In this method, seven layers of fiber were used. The precut fibers were placed into the mold, epoxy was poured onto them, and a metal roller was employed to ensure uniform distribution of the resin over the fiber layers. After repeating this process for each layer, the mold was closed and left to cure for 24 h at room temperature. Subsequently, the cured composite laminate was removed from the mold and subjected to a post-cure treatment at 100 °C for 4 h.

### 2.2. Experimental Setup

**Figure 2:**
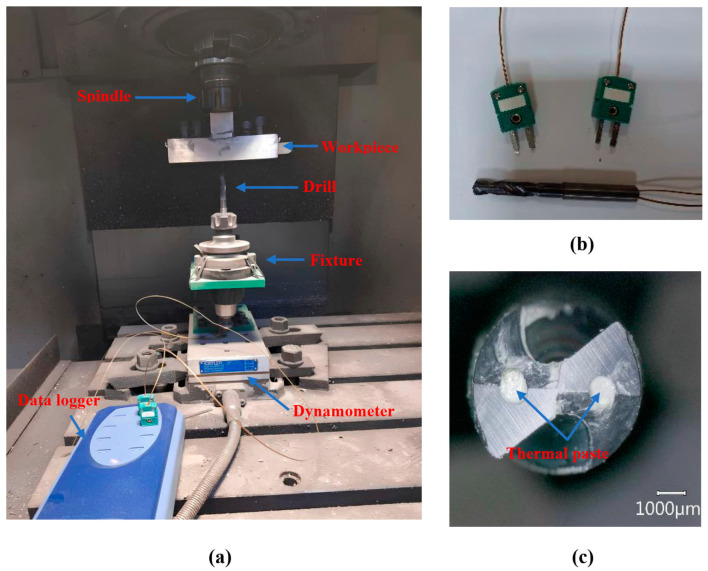
(**a**) Experimental setup, (**b**) placement of thermocouples near the drill tip, (**c**) application of thermal paste inside the cooling channels.

Drilling experiments were conducted under dry cutting conditions using a Quaser MV 154C CNC vertical machining center (Quaser Machine Tools, Taichung City 437106, Taiwan). A specially designed cutting tool (code: 460.1-0680-020A1-XM GC34) with a diameter of 6.8 mm was employed for drilling the composite materials. The tool features a point angle of 140° and is coated with TiAlN using the PVD method (Figure 2). For each of the five different specimens, drilling tests were performed using spindle speeds of 1500 and 3000 rpm and feed rates of 50, 75, and 100 mm/min (Table 3).

The composite materials were manufactured in the form of laminated plates, and one plate was produced for each material type. For each material–parameter combination, drilling experiments and corresponding measurements were repeated three times to ensure repeatability. The reported thrust force, cutting temperature, and surface roughness values represent the average of these repeated measurements.

The drilling process was carried out using a reverse-fixture setup, in which the cutting tool remains stationary while the work piece is in motion. The drill bit was securely mounted onto the force dynamometer, and the composite materials were clamped into a specially manufactured fixture. This fixture ensured rigid attachment of the work piece to the spindle. During drilling, thrust force and temperature were measured and recorded to investigate the influence of machining parameters.

The thrust force generated during drilling was measured using a Kistler 9257B dynamometer (Kistler Instrumente AG, Winterthur, Switzerland). Cutting temperatures were collected in real-time through K-type thermocouples (with a cable diameter of 0.8 mm and a wire diameter of 0.5 mm) strategically positioned to ensure direct contact at the tool–workpiece interface. To capture temperatures as close to the primary cutting zone as possible, the thermocouples were routed through the tool’s internal passages and fixed at the tip to maintain constant contact during engagement. In addition, the gap between the cooling channel and the probe was filled with thermal paste. The sensors provided a measurement accuracy of ±0.4%, and the data were transferred to a computer via a high-speed data logger to capture rapid thermal gradients (Figure 2).

## 3. Results and Discussions

### 3.1. Analysis of the Thrust Force

**Figure 3:**
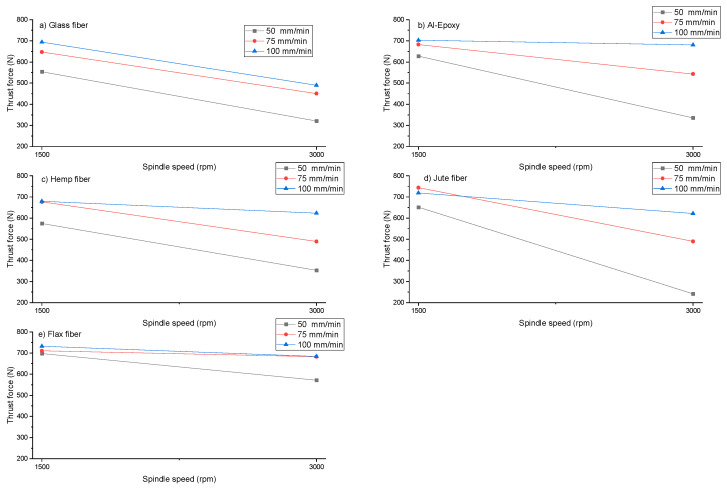
Effect of different cutting parameters on thrust force during drilling of (**a**) Glass Fiber, (**b**) Al–epoxy, (**c**) Hemp Fiber, (**d**) Jute Fiber, and (**e**) Flax Fiber specimens.

Industrial and academic studies have demonstrated that delamination damage occurs when the thrust force applied during drilling exceeds the interlaminar strength of the composite. This threshold is commonly referred to in the literature as the critical thrust force [20]. Therefore, accurate analysis of thrust force is essential for assessing the machinability of composites and minimizing drilling-induced damage. In this study, the maximum thrust force data recorded during the drilling of five different composite materials under varying cutting parameters were plotted and are presented in Figure 3. The results indicate that thrust force depends not only on the type of material but also on the applied machining parameters, with each composite exhibiting distinct behavior.

As shown in Figure 3, an increase in feed rate leads to higher thrust forces across all composite specimens, whereas an increase in spindle speed reduces the thrust force. This trend is consistent with previously reported results in the literature [21]. The reduction in thrust force at higher spindle speeds is attributed to the softening effect of elevated cutting zone temperatures on the epoxy matrix, which reduces cutting resistance [6]. Conversely, increasing the feed rate raises the volume of material removed per unit time and increases the load on the tool, resulting in higher cutting forces. This highlights the trade-off between machining efficiency and damage control, as higher feed rates accelerate material removal but also increase the risk of delamination.

When comparing different fiber reinforcements, similar thrust force values were observed for the synthetic specimens (glass fiber and Al–epoxy). For the glass fiber-reinforced specimen, thrust forces ranged between 320 and 693 N, while for the Al–epoxy specimen, they ranged between 335 and 702 N. The Al–epoxy specimen exhibited approximately slightly 10–15 N higher thrust forces than the glass fiber-reinforced specimen. This behavior differs from commonly reported trends in which fiber reinforcement may increase cutting resistance, indicating that the effect of reinforcement on thrust force is strongly dependent on the reinforcement type and matrix characteristics. Notably, under the investigated drilling conditions, the Al–epoxy specimen exhibited slightly higher peak thrust force values than the glass fiber-reinforced composite. This underscores that peak thrust force is not solely determined by the presence of fiber reinforcement. In this study, the epoxy was reinforced with aluminum particles, and the Al–epoxy specimen contained a higher proportion of aluminum particles than the other specimens, which can explain the higher thrust forces observed. This suggests that particulate reinforcement in the matrix can have a more dominant effect on thrust forces than fiber reinforcement alone, particularly when fiber content is relatively low.

The thrust force, serving as a primary indicator of drilling resistance for the composites studied, can be explained through the interaction of two fundamental mechanisms: (1) the cutting/crushing of the Al-reinforced epoxy matrix and friction-induced resistance, and (2) the shearing or breaking of the fibers, alongside debonding and pull-out at the fiber-matrix interface. The findings demonstrate that drilling resistance, particularly in terms of peak thrust force, cannot be attributed solely to the presence of fibers but is governed by the combined effects of matrix properties, reinforcement type, and filler content. Especially when high Al-filler content is used, the structural and tribological properties of the matrix—such as hardness, brittleness, and abrasiveness—play a decisive role in thrust force generation. The fact that the Al–epoxy specimens without fiber reinforcement exhibited slightly high thrust forces indicates that the first factor significantly influences the overall resistance. High concentrations of Al particles may limit the matrix’s deformation capability, shifting the material removal mechanism toward a crushing-fracturing character rather than pure cutting. Furthermore, Al particles can increase tool abrasion, reducing cutting efficiency while increasing the frictional component and, consequently, the thrust force. Therefore, the higher thrust force observed in the Al–epoxy specimen compared to the glass fiber-reinforced counterpart highlights the dominant role of particle loading over the absolute effect of fiber reinforcement.

For the natural fiber-reinforced composites, thrust forces during drilling of the hemp specimen ranged from 352 to 679 N, for the jute specimen from 241 to 719 N, and for the flax specimen from 571 to 732 N. Among all specimens, the highest thrust force was measured for the flax specimen, while the lowest was recorded for the jute specimen. The generally higher cutting forces observed in natural fiber-reinforced composites are attributed to the heterogeneous and lignocellulosic nature of the fibers, which can lead to increased fiber pull-out and matrix separation during cutting. This behavior underscores the challenge of machining natural fiber composites, where fiber morphology and interfacial bonding play critical roles in determining force requirements, delamination risk, and ultimately the quality of the drilled hole.

**Figure 4:**
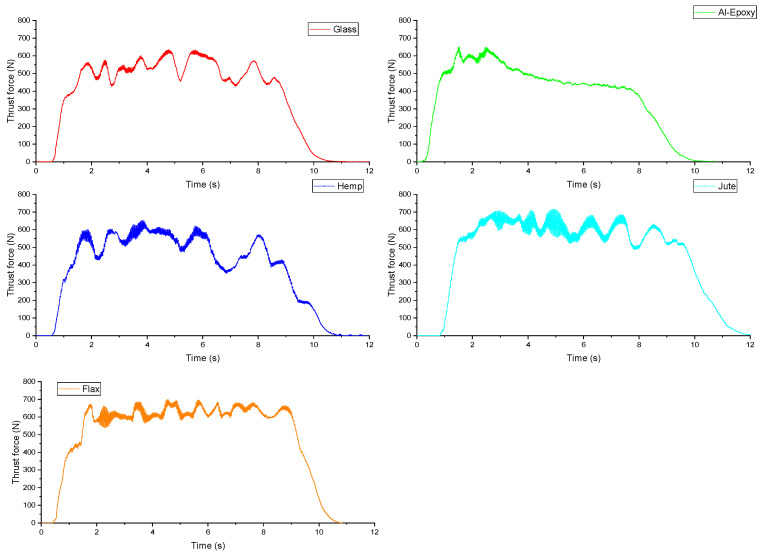
Thrust force curves obtained during tests at a spindle speed of 1500 rpm and a feed rate of 75 mm/min.

Time-dependent variations in thrust force during drilling for different specimens under a spindle speed of 1500 rpm and a feed rate of 75 mm/min are presented in Figure 4. Unlike the other specimens, the Al–epoxy specimen exhibited a high initial thrust force at drill entry, followed by a decreasing trend with increasing hole depth. In all other specimens, fluctuating thrust forces were observed throughout the drilling process. These fluctuations are attributed to the heterogeneous and layered structure of the materials, caused by the fiber reinforcements. The observed variations reflect intermittent fiber–matrix failure and varying local resistance within the composite, which is more pronounced in natural fiber-reinforced specimens due to their anisotropy and irregular fiber distribution. In addition, higher thrust forces correlate with elevated cutting temperatures, which can further soften the matrix and exacerbate delamination at the hole entry and exit. Therefore, thrust force is not only an indicator of cutting resistance but also a predictive parameter for thermal effects and potential hole quality deterioration.

These observations further emphasize that peak thrust force and overall force evolution during drilling represent different aspects of machining behavior and should be interpreted separately.

### 3.2. Evaluation of Temperature Measurements

The temperature generated during drilling is a critical factor that directly influences output parameters such as tool–material interaction damage, surface roughness, delamination, and tool wear. Consequently, analyzing temperature variations is essential for evaluating the machinability of composites. In this study, cutting zone temperature values were recorded in real time for all specimens, and the resulting thermal profiles were assessed with respect to fiber type, fiber–matrix interface quality, and the thermal conductivity characteristics of each material.

**Figure 5:**
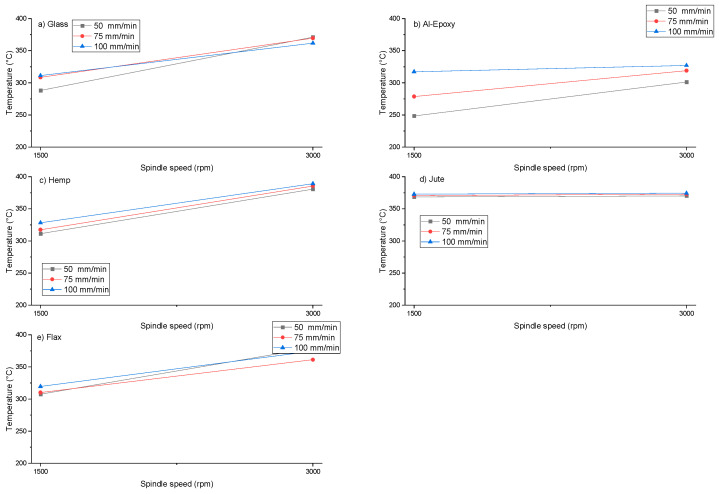
Maximum cutting temperatures measured during drilling of (**a**) Glass Fiber, (**b**) Al–epoxy, (**c**) Hemp Fiber, (**d**) Jute Fiber, and (**e**) Flax Fiber composites.

The primary focus of this investigation is to examine the cutting forces and thermal response during drilling of natural fiber-reinforced composites prepared using an epoxy matrix reinforced with aluminum particles. Figure 5 presents the maximum temperatures measured during drilling as a function of fiber reinforcement type and cutting parameters. Examination of the figure reveals significant differences in cutting temperatures between natural and synthetic fiber-reinforced composites, which vary according to the applied machining conditions.

Within the scope of the experiments, it was observed that increases in both feed rate and spindle speed generally led to higher cutting zone temperatures for all composite specimens. This trend aligns with previous reports in the literature [22]. Among the natural fiber composites, the jute specimen exhibited the most uniform temperature profile, with closely clustered values across all tests, suggesting a consistent thermal response during drilling. This may be linked to its relatively homogeneous fiber distribution and lower fiber stiffness, which reduce abrupt fluctuations in cutting resistance and local heat generation.

When comparing reinforcement types, synthetic specimens (glass fiber and Al–epoxy) showed similar temperature ranges. For the glass fiber-reinforced specimen, temperatures ranged from 288 to 371 °C, whereas the Al–epoxy specimen recorded temperatures between 248 and 327 °C. Notably, the Al–epoxy specimen exhibited approximately 40–45 °C lower cutting zone temperatures than the glass fiber composite. This difference may be attributed to the smoother surface of the epoxy matrix, which results in lower frictional resistance at the tool–workpiece interface and reduced heat generation. Furthermore, the absence of fibers that act as a thermal insulator in the Al–epoxy specimens, combined with the high concentration of aluminum particles, significantly alters the thermal behavior of the material. In this configuration, the aluminum particles act as high-efficiency thermal sinks, rapidly conducting generated heat away from the cutting zone and dissipating it into the bulk of the material. Consequently, despite the higher energy consumption indicated by elevated thrust forces, this rapid heat dissipation results in lower peak temperatures at the tool-workpiece interface compared to fiber-reinforced counterparts.

For the natural fiber-reinforced composites, cutting temperatures ranged from 311 to 389 °C for the hemp specimen, 368 to 374 °C for the jute specimen, and 307 to 379 °C for the flax specimen. Among all specimens, the highest temperatures were observed in the hemp composite, while the lowest were recorded for the Al–epoxy specimen. Overall, natural fiber composites exhibited higher cutting temperatures than synthetic composites. This is primarily due to the surface characteristics and microstructural features of natural fibers, which enhance mechanical interlocking with the aluminum particle-reinforced epoxy matrix [23]. The increased mechanical resistance during material removal results in elevated cutting forces, which in turn generate higher temperatures in the cutting zone. In contrast, synthetic glass fibers, having a smoother surface and more uniform geometry, create weaker mechanical interlocking with the matrix, reducing cutting resistance and heat generation.

During drilling, the measured temperatures in the range of 300–390 °C exceed the typical degradation thresholds of epoxy matrices. However, these values correspond to highly localized and transient contact temperatures at the tool–workpiece interface rather than the bulk temperature of the composite. The duration of these peak temperatures is extremely short, and the thermal exposure is confined to a very thin region adjacent to the drilled hole. Moreover, heat is rapidly dissipated into the surrounding material and partially removed with the generated chips, preventing a significant temperature rise in the bulk composite. Although localized surface-level matrix softening or micro-scale thermal damage may occur, the overall structural integrity of the composite remains unaffected during the drilling process.

The measured thermal behavior also reflects differences in thermo-mechanical response among the natural fiber composites. The rougher surfaces and heterogeneous nature of hemp and flax fibers provide a higher resistance to cutting, leading to localized heating. However, this same interfacial strength helps maintain structural integrity, preventing sudden material failure and enabling a more balanced distribution of thermal energy. In contrast, the relatively uniform structure of jute fibers results in a more consistent temperature profile but slightly lower maximum temperatures, reflecting its lower resistance to material removal.

The observed differences in drilling temperature among the composites can be primarily attributed to variations in their thermal conductivity. Although the thermal conductivity of the composites was not directly measured in this study, the interpretation is based on the well-established intrinsic thermal properties of the constituent materials.

The Al–epoxy composite contains a high aluminum filler loading (60 vol.%), which promotes particle-to-particle contact within the epoxy matrix. This interconnected filler network significantly enhances heat transfer through the composite, allowing heat generated at the tool–workpiece interface to be rapidly conducted into the bulk material. Consequently, the Al–epoxy composite behaves as an effective heat sink, resulting in lower localized temperatures during drilling.

In contrast, natural fiber reinforced epoxy composites exhibit comparatively lower thermal conductivity due to the insulating nature of natural fibers and the presence of fiber–matrix interfaces, which impede heat flow. This reduced heat dissipation capability leads to localized heat accumulation at the tool–workpiece interface, thereby increasing the measured drilling temperatures.

**Figure 6:**
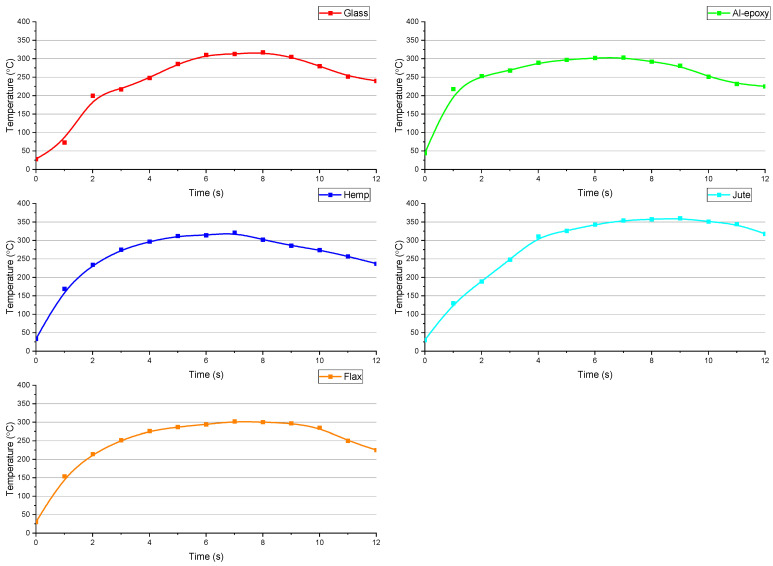
Temperature curves obtained during tests at a spindle speed of 1500 rpm and a feed rate of 75 mm/min.

Figure 6 illustrates the cutting zone temperature curves obtained during drilling at a spindle speed of 1500 rpm and a feed rate of 75 mm/min. For the glass fiber composite, the temperature increased gradually, whereas the Al–epoxy specimen exhibited a sharp temperature spike upon initial tool–material contact. The natural fiber composites, by comparison, displayed a more moderate temperature increase, following a parabolic trend. This indicates that natural fiber composites, particularly hemp and flax, exhibit superior thermo-mechanical behavior under the tested conditions, as the gradual temperature rise reduces the risk of localized thermal damage, such as matrix softening, fiber degradation, or delamination. The sudden temperature spikes observed in the Al–epoxy specimen, on the other hand, suggest that even small increases in cutting forces can lead to abrupt heat generation, which may compromise hole quality and matrix stability.

These findings demonstrate that natural fiber-reinforced composites exhibit a more balanced thermo-mechanical response compared to synthetic composites when reinforced within an aluminum particle–epoxy matrix. The interplay between fiber morphology, fiber–matrix adhesion, and machining parameters determines the thermal behavior, which in turn affects cutting forces, damage formation, and surface integrity. These results highlight the importance of optimizing drilling conditions to control cutting zone temperature, improve hole quality, and minimize thermally induced damage in natural fiber composites.

### 3.3. Evaluation of Hole Exit Damage

**Figure 7:**
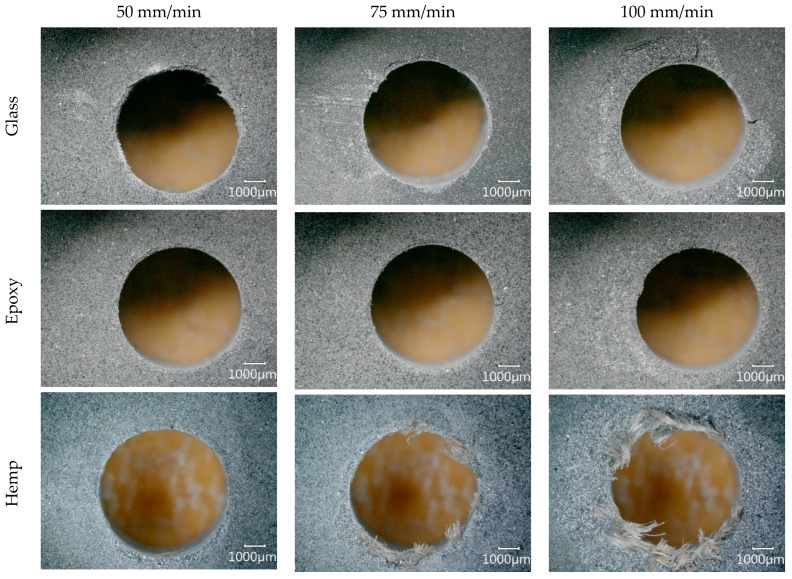

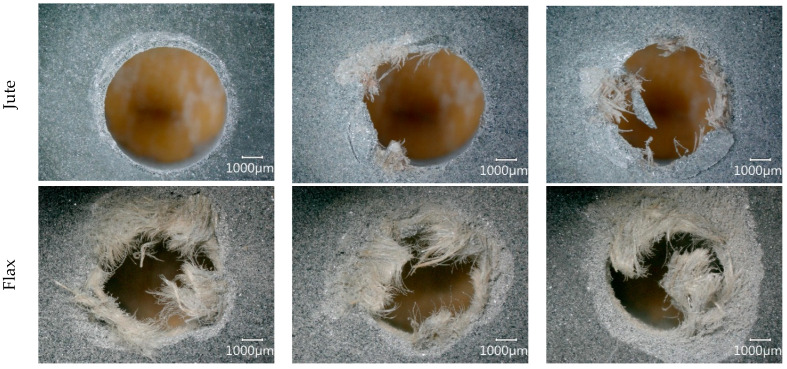
Hole exit images obtained during tests at a spindle speed of 1500 rpm.

When composites with a layered structure are drilled, interlaminar separation, commonly referred to as delamination, can occur due to the effect of thrust force. This phenomenon is particularly pronounced at the hole exit, where the material is no longer supported by the substrate. Therefore, evaluating exit-hole damage after drilling is critical for assessing composite machinability and ensuring structural integrity. In this study, the exit-hole damages of five different composite materials drilled at varying feed rates were captured using a digital microscope, and the results are presented in Figure 7 (low spindle speed, 1500 rpm) and 8 (high spindle speed, 3000 rpm).

The images indicate that increasing the feed rate generally resulted in more severe exit-hole damage across all specimens. The hole exits of the glass fiber-reinforced and Al–epoxy composites were noticeably cleaner than those of the natural fiber-reinforced materials, suggesting smoother cutting and lower material separation. In contrast, the natural fiber-reinforced composites exhibited a pronounced presence of uncut fibers at the hole exit. This difference can be attributed to the more stable fiber–matrix interface in synthetic composites and the comparatively lower cutting zone temperatures, which reduce thermal softening and material pull-out.

**Figure 8:**
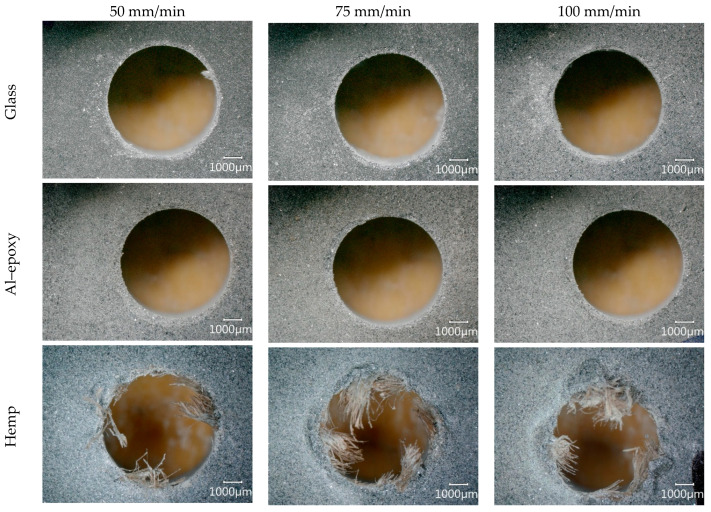

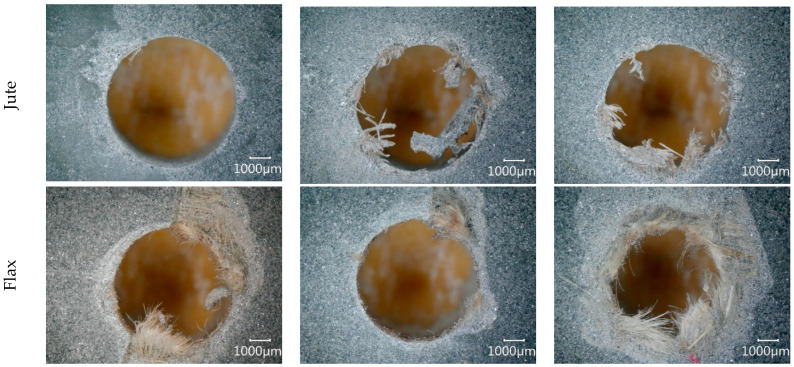
Hole exit images obtained during tests at a spindle speed of 3000 rpm.

Among all specimens, Al–epoxy displayed the cleanest and least damaged hole exit. In the glass fiber-reinforced composite, severe exit-hole damage was observed under the cutting condition of 1500 rpm and 100 mm/min (Figure 7), whereas higher spindle speed conditions (3000 rpm) minimized the damage (Figure 8). This indicates that spindle speed optimization can play a key role in mitigating exit-hole defects, likely due to reduced thrust force fluctuations and localized heating effects.

In natural fiber-reinforced composites, particularly the flax specimen, a substantial presence of uncut fibers was consistently observed at the hole exit, indicating that a high-quality cutting process was not achieved. This observation is closely related to the higher thrust forces generated during drilling, suggesting that natural fiber-reinforced composites generally exhibit lower machinability compared to synthetic-fiber-reinforced materials. The heterogeneous structure and lignocellulosic nature of natural fibers are likely to contribute to this behavior by increasing material resistance and promoting fiber pull-out.

When individual natural fiber composites are considered, fewer uncut fibers were observed in the hemp composite at low spindle speeds, indicating better hole exit quality. However, as spindle speed increased, the amount of uncut fibers also rose, likely due to the corresponding increase in cutting zone temperature (Figure 5c), which may soften the matrix and reduce interfacial cohesion. Except for the condition of 1500 rpm and 50 mm/min, most holes in natural fiber composites required secondary operations to improve exit quality, highlighting the challenge of achieving acceptable hole integrity in these materials without post-processing.

For the jute fiber-reinforced composite, no clear correlation between spindle speed and the formation of uncut fibers was observed. High feed-rate drilling consistently produced a substantial number of uncut fibers at both spindle speeds, while low feed-rate conditions (1500 rpm, 50 mm/min) minimized fiber pull-out and produced a more uniform damage distribution around the hole edges. As the feed rate increased, cracks propagated from the hole edges into the surrounding material, reflecting the combined effects of increased thrust force and material heterogeneity.

For the flax fiber-reinforced composite, drilling at 1500 rpm resulted in similar exit-hole appearances across all feed rates, with significant fiber pull-out and delamination. Holes produced under these conditions would require secondary operations to achieve functional quality. Increasing spindle speed slightly reduced the number of uncut fibers, but substantial exit-hole damage persisted. Among the natural fiber-reinforced specimens, the flax composite displayed the worst exit-hole quality, characterized by both severe delamination and widespread fiber protrusion.

The high thrust forces measured during drilling of the flax specimen (Figure 3e), combined with elevated cutting zone temperatures observed in natural fiber composites, can be directly linked to the observed exit-hole damage. Elevated temperatures reduce the matrix’s resistance to deformation and promote interlaminar separation, while high thrust forces exacerbate material delamination and propagate damage over a larger area. This combination of mechanical and thermal effects explains the particularly poor exit-hole quality observed in the flax specimen and underscores the importance of optimizing drilling parameters to minimize damage in natural fiber composites.

The results highlight a clear relationship between fiber type, machining parameters, and hole exit integrity. Synthetic-fiber and fiber-free composites exhibit better exit-hole quality due to lower cutting forces and reduced thermal effects, while natural fiber composites show increased susceptibility to delamination and uncut fibers, particularly under high feed-rate or elevated spindle-speed conditions. These findings emphasize the need for careful selection of feed rate, spindle speed, and potentially secondary finishing operations to achieve acceptable hole quality in natural fiber-reinforced composites.

### 3.4. Evaluation of Inner Hole Surface Roughness

**Figure 9:**
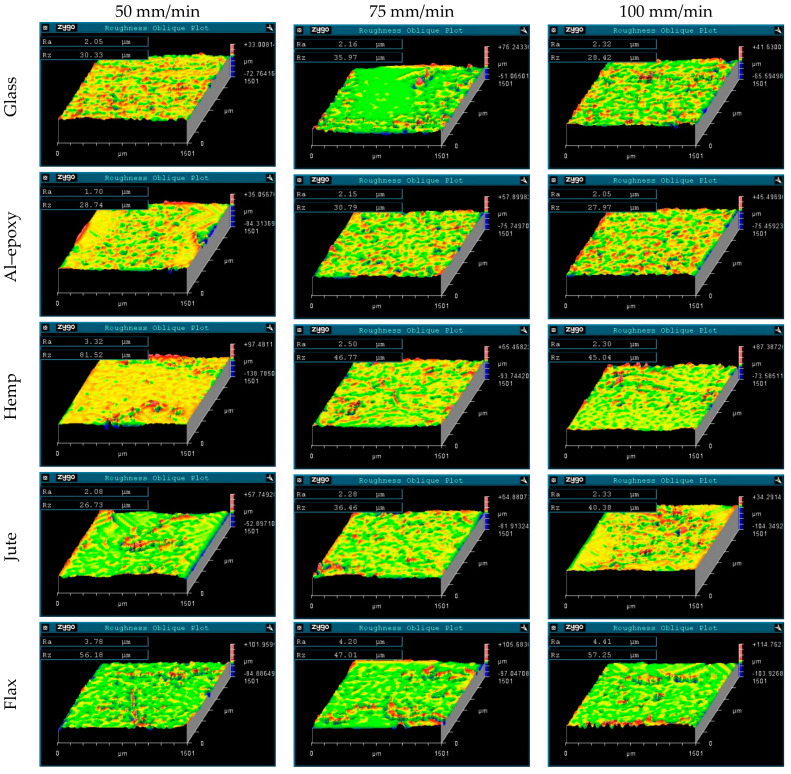
Inner Hole surface morphologies obtained during tests at a spindle speed of 1500 rpm.

**Figure 10:**
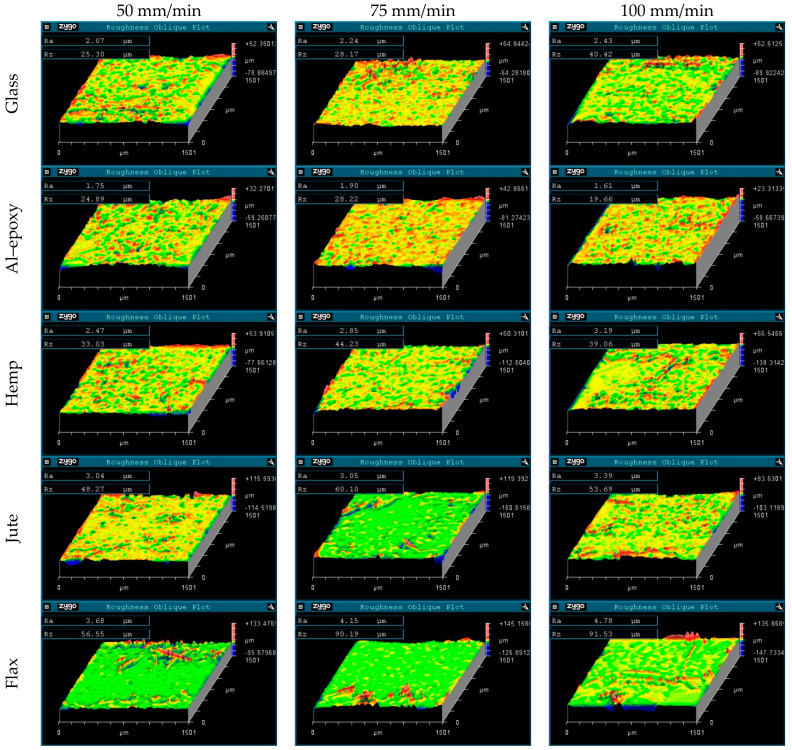
Inner hole surface morphologies obtained during tests at a spindle speed of 3000 rpm.

After evaluating the hole exit damage, the drilled specimens were sectioned along their axes, and the inner hole surfaces were examined using an optical profiler. The inner hole surface morphologies for tests conducted at a spindle speed of 1500 rpm are presented in Figure 9, while those for tests at 3000 rpm are shown in Figure 10. This analysis provides a more detailed understanding of hole quality and allows for correlation with both exit-hole damage and drilling parameters.

For the glass fiber-reinforced composite, an increase in feed rate led to a deterioration in hole quality, as indicated by higher surface roughness (*Ra*). In contrast, increasing the spindle speed did not significantly affect the hole surface finish, with *Ra* values remaining similar to those measured at the lower spindle speed. This behavior differs from previous reports, which suggested higher surface roughness at low spindle speeds during composite drilling [24,25]. The deviation observed in this study can be attributed to the aluminum particle-reinforced epoxy matrix, where higher cutting temperatures likely influence the surface morphology by softening the matrix and altering the chip formation mechanism.

For the epoxy composite, no clear linear relationship between cutting parameters and *Ra* values was observed, indicating that the inner hole surface quality is less sensitive to the spindle speed and feed rate within the tested parameter range. This may be due to the more homogeneous matrix structure and the absence of fibrous reinforcements, which reduces local heterogeneity and micro-defects that typically affect surface roughness.

In natural fiber-reinforced composites, distinct behaviors were observed depending on the fiber type. For the hemp fiber-reinforced composite, inner hole surface roughness showed no clear correlation with feed rate at 1500 rpm. However, at a spindle speed of 3000 rpm, higher feed rates led to a deterioration in hole quality, reflected by increased *Ra* values. This can be attributed to elevated cutting temperatures, which promote fiber degradation and matrix softening, thereby increasing surface irregularities [26].

For the jute fiber-reinforced composite, increasing the feed rate consistently worsened the hole surface finish, while higher spindle speeds did not improve the quality; in fact, *Ra* values tended to increase at elevated spindle speeds. Similarly, the flax fiber-reinforced composite displayed higher surface roughness with increasing feed rate. At low feed rates, an increase in spindle speed slightly improved the inner hole surface quality, suggesting that moderate cutting speeds may promote smoother chip evacuation without excessive thermal softening.

Comparing fiber reinforcements, the lowest surface roughness was measured in the Al–epoxy specimen, followed by the glass, hemp, and jute specimens, with the highest roughness observed in the flax specimen. Notably, the elevated *Ra* values for the flax specimen are consistent with the pronounced exit-hole damage observed in this material, demonstrating a direct correlation between hole exit defects and the condition of the inner hole surface. This observation also indicates that visual inspection of exit-hole damage can serve as a preliminary indicator of inner hole surface quality.

Overall, natural fiber-reinforced composites exhibited higher *Ra* values compared to synthetic specimens, reflecting the influence of fiber heterogeneity and microstructural complexity. The Al–epoxy specimens showed a more uniform and regular surface topography, whereas natural fiber composites displayed increased irregularities due to micro-scale defects such as fiber pull-out, debonding, and fiber protrusion. Glass fiber-reinforced composites exhibited smoother surfaces relative to natural fiber composites, highlighting the positive effect of the homogeneous and continuous structure of synthetic fibers on machinability and inner hole surface quality. These findings emphasize that fiber type, spindle speed, and feed rate are critical factors influencing the surface integrity of drilled composite materials, and careful optimization of these parameters is essential to achieve acceptable hole quality in both synthetic and natural fiber-reinforced composites.

The present study provides a comprehensive experimental evaluation of drilling behavior, cutting zone temperatures, thrust forces, hole exit damage, and inner hole surface roughness in natural fiber, glass fiber, and Al–epoxy composites. Experiments were conducted under controlled dynamic drilling conditions at selected spindle speeds and feed rates, enabling detailed analysis of the influence of fiber type and cutting parameters on the drilling response. However, several limitations exist that can guide future research. The investigation was limited to specific spindle speeds, feed rates, and a small set of fiber types, tool geometries, and cutting conditions, which may not capture the full spectrum of industrial drilling scenarios. Additionally, while the authors attempted to simulate the drilling processes, the necessary material constants for the aluminum particle-reinforced epoxy were not available and have not yet been experimentally characterized; therefore, accurate numerical modeling of these composites remains incomplete. Furthermore, the study focused on short-term thermal and mechanical responses and did not consider potential long-term effects, such as residual stresses or fatigue induced by drilling, nor were parameters such as tool wear, microstructural matrix changes, or fiber degradation assessed. Future work will aim to expand the parameter space by including a wider range of spindle speeds, feed rates, tool geometries, coating strategies, and cooling/lubrication conditions, as well as different fiber orientations, hybrid fiber composites, and multi-layered configurations. Once the material properties of the aluminum particle-reinforced epoxy are fully characterized, validated finite element models and predictive simulations can be developed to provide deeper insights into the parameter–temperature–damage relationships, enabling optimized drilling strategies and broader applicability in industrial settings.

### 3.5. Evaluation of Bore Hole

**Figure 11:**
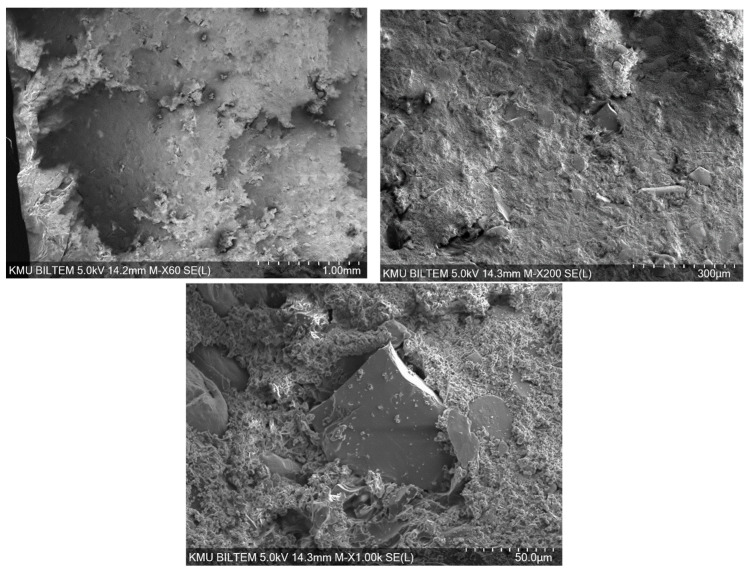
Bore-hole damage to the epoxy/aluminum sample.

The SEM images indicate that the aluminum particles used as reinforcement exhibit non-uniform shapes and distribution; partial agglomerations are present due to the 60% volume loading (Figure 11). In some regions, good wetting of the reinforcement by the Al–epoxy and strong interfacial bonding are observed, whereas in other regions interfacial voids and particle pull-out traces suggest local adhesion weakness. This heterogeneity can reduce mechanical performance in poorly bonded areas, while increasing the risk of localized stiffness rise and embrittlement around agglomerated regions.

**Figure 12:**
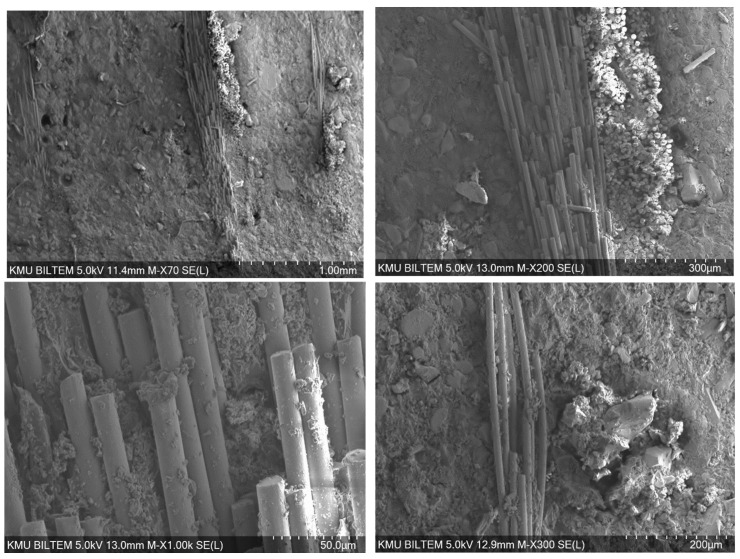
Bore-hole damage to the epoxy/aluminum/glass fiber.

The SEM images of the epoxy composite reinforced with glass fiber fabric and containing a high fraction of Al particles (60%) point to a distinctly heterogeneous microstructure (Figure 12). Because 60% Al is a very high loading, the viscosity of the epoxy increases substantially. This hinders resin flow between glass fiber bundles and elevates the risk of voids/porosity and particle clustering (agglomeration). The images suggest that the restricted matrix flow associated with the increased viscosity prevents complete impregnation. In addition, Al particle agglomerates and irregular clusters accompanied by micro-voids are observed, which may be related to weak wetting (adhesion) in certain areas. The fiber–matrix interface appears weakened in some regions by interfacial gaps, while in other regions limited adhesion is preserved, as evidenced by partial matrix residues on the fibers. Fiber pull-out traces around agglomerates, together with interfacial weakness and stress concentration, are also apparent.

**Figure 13:**
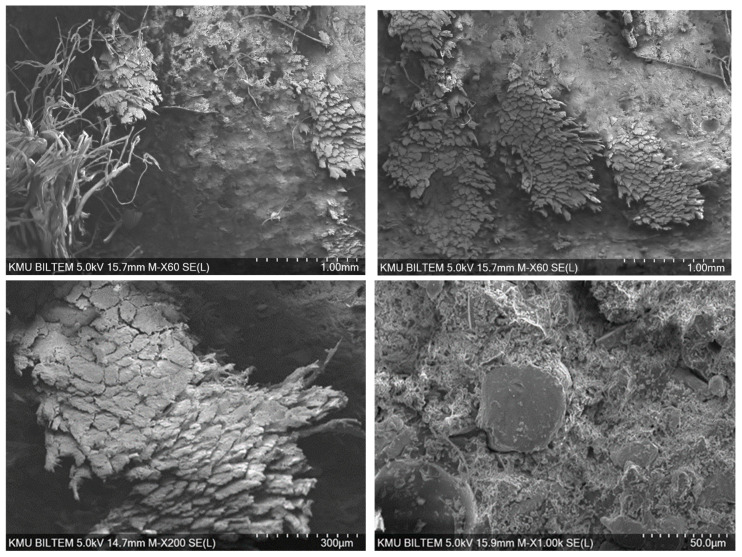
Bore-hole damage to the epoxy/aluminum/jute fiber.

The SEM images show that, in the jute-fiber-reinforced composite containing 60% Al particles in the epoxy matrix, damage is shaped particularly by the bundle structure of jute and by the heterogeneity of the natural fiber–matrix interface (Figure 13). Fiber bundle separation, pull-out and fraying (fibrillation) at fiber ends, and traces of weak adhesion in some regions are prominent on the fracture surface. These features can be evaluated together with impregnation difficulties caused by the viscosity increase induced by the high filler content. Local particle clustering and resin-starved areas around the fibers indicate the coexistence of agglomeration-induced stresses and weak fiber–matrix regions.

**Figure 14:**
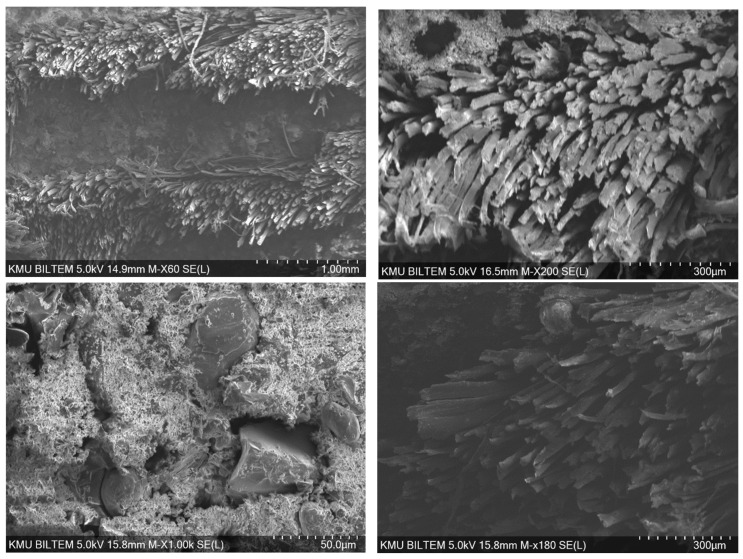
Bore-hole damage to the epoxy/aluminum/flax fiber.

The SEM images reveal a clearly heterogeneous microstructure in the hybrid composite where flax fiber fabric is used together with Al particles at a high filler loading (60%) in the epoxy matrix (Figure 14). Resin-weak regions around flax bundles and fracture traces following bundle boundaries suggest that the elevated viscosity associated with the high particle content restricts matrix flow and leads to non-uniform impregnation. The tendency of Al particles to form local clusters/agglomerates and the potentially micro-voided brittle fracture texture around these particles can be considered critical zones that may act as stress concentration sites. Compared with jute-reinforced composites, the presence of limited matrix residues on the flax fiber surface in some areas suggests partial adhesion, whereas cleaner fiber surfaces and interfacial discontinuities in other areas indicate local weak bonding. Together with the bundle-based natural fiber morphology of flax, this interfacial heterogeneity suggests that both fiber pull-out and bundle separation, accompanied by agglomeration-related stress, may play a role in the fracture mechanism and limit load-transfer efficiency.

**Figure 15:**
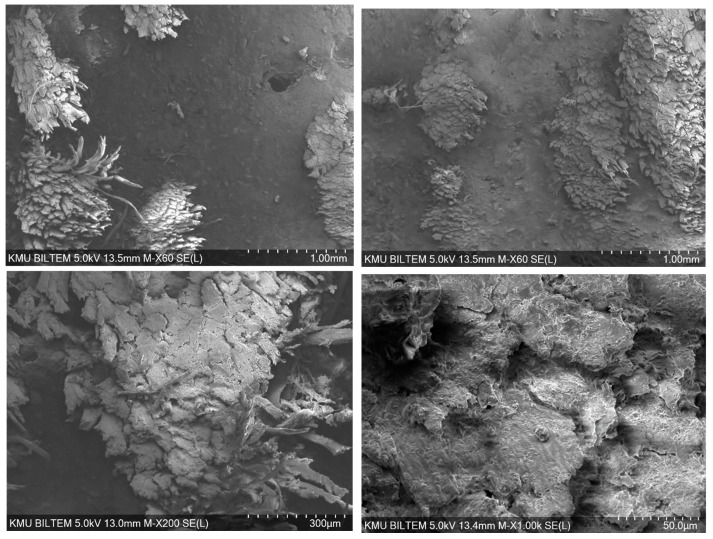
Bore-hole damage to the epoxy/aluminum/hemp fiber.

Among natural fibers, hemp is characterized by thick/stiff bundle structures and a pronounced tendency to align. Therefore, the fracture surface often displays more linear traces following bundle boundaries (Figure 15), giving hemp a character close to flax. In some regions, matrix residues on the fiber surface (partial mechanical interlocking) are observed, whereas interfacial gaps (weak bonding) appear in others. With 60% Al, particle agglomerates and resin-starved zones become especially evident around the fiber bundles. When combined with the already “bundle-oriented” microstructure of hemp, this can strengthen the dual effect of weak interfaces and agglomeration-induced stress formation, potentially accelerating crack propagation.

While more dispersed bundle separation and fibrillation (fraying) are prominent in jute, fracture in hemp is more strongly governed by the alignment of thick bundles and bundle-boundary-controlled interfacial behavior. Due to Al-particle agglomeration and local resin deficiency, these regions become more brittle.

The SEM images collectively demonstrate that all glass fiber, jute, flax, and hemp fabric-reinforced epoxy composites containing a high Al particle loading (60%) share common microstructural consequences of increased viscosity: heterogeneous morphology, local particle clustering leading to agglomeration, and locally limited impregnation around fibers. Nevertheless, fracture characteristics differ markedly depending on fiber type. In the glass fiber-reinforced system, the fracture surface forms a relatively “cleaner” interface; damage is mainly associated with stresses originating from particle agglomerates and locally weak fiber–matrix adhesion. In natural fiber composites, fracture behavior is largely guided by bundle morphology. In jute reinforcement, bundle separation, pronounced pull-out, and fibrillation at fiber ends dominate, whereas in flax reinforcement crack propagation tends to follow more linear and orderly paths along bundle boundaries and pull-out appears relatively limited. The hemp-reinforced system similarly shows bundle-oriented cracking akin to flax, yet due to thicker bundle structures it gives the impression of a more “blocky” separation and bundle-level interfacial heterogeneity. These findings indicate that 60% Al filler increases brittleness and the sensitivity of the interface/impregnation in all systems; meanwhile, in natural fibers the damage path differentiates along the jute–flax–hemp line as a function of bundle morphology. Accordingly, improving both particle dispersion and fiber–epoxy interfacial compatibility through appropriate surface treatments and effective mixing strategies is critical.

Although the same epoxy matrix and identical aluminum particle reinforcement were used for all composite systems to ensure comparable processing conditions, it should be noted that material quality in fiber-reinforced composites is influenced by multiple factors beyond matrix formulation. Parameters such as fiber volume fraction, fiber dispersion and orientation, void content, and fiber–matrix interfacial quality may vary among different fiber systems and were not independently quantified in the present study.

Therefore, the observed differences should be interpreted as the combined effect of fiber type and composite architecture rather than as purely isolated fiber type effects. In addition, the presence of aluminum particle reinforcement introduces additional matrix–particle and particle–fiber interfaces, which may interact differently with natural and glass fibers and influence local load transfer and thermal–tribological behavior during drilling. While a detailed interfacial characterization is beyond the scope of this study, the experimental design ensures consistent fabrication procedures across all specimens, allowing meaningful comparative analysis under identical processing and machining conditions.

## 4. Conclusions

In this study, the drilling behavior of five composite systems—three natural fiber-reinforced composites (jute, flax, and hemp) with aluminum particle-reinforced epoxy matrices, and a glass fiber-reinforced composite—were evaluated under varying cutting parameters. Cutting zone temperatures and thrust forces were recorded using embedded thermocouples and a dynamometer, while hole exit damage and inner hole surface roughness were assessed to characterize machining quality. The main findings are summarized as follows:

- Increasing spindle speed led to a reduction in thrust forces, primarily due to thermal softening of the aluminum particle-reinforced epoxy matrix, whereas higher feed rates increased thrust forces because of the greater material removal per unit time.
- Natural fiber-reinforced composites consistently exhibited higher thrust forces than the synthetic (glass fiber) and fiber-free epoxy specimens. This trend is attributed to stronger mechanical interlocking at the natural fiber–matrix interface.
- Cutting zone temperatures increased with both spindle speed and feed rate. Among all tested materials, the hemp fiber composite exhibited the highest temperature rise, while the Al–epoxy produced the lowest temperatures.
- Among the natural fibers, flax fiber-reinforced composites showed a balanced machining response, displaying moderate temperature rise despite comparatively high thrust forces. In contrast, jute and hemp composites exhibited higher temperature generations due to differences in interfacial bonding characteristics.
- Natural fiber-based composites exhibited more pronounced hole exit damage and poorer inner hole surface quality compared to the glass fiber composite. Among the natural fibers, the flax composite experienced the most severe exit damage and highest surface roughness.
- At a spindle speed of 1500 rpm, the measured average surface roughness (Ra) values ranged from 2.05 to 2.32 µm for the glass fiber-reinforced specimen, 1.70 to 2.15 µm for the epoxy specimen, 2.30 to 3.32 µm for the hemp specimen, 2.08 to 2.33 µm for the jute specimen, and 3.78 to 4.41 µm for the flax specimen.

## Figures and Tables

**Table 1:** Properties of the fibers and produced composites.

Matrix Type	Fiber Type	Fiber Orientation	Number of Layers	Fiber Fabric Weight	Thickness (mm)	Fiber Weight Ratio (%)
Epoxy + Al	–	–	–	–	11	-
Epoxy + Al	Jute	0/90°	7	270 g/m^2^	11	9.9
Epoxy + Al	Flax	0/90°	7	300 g/m^2^	11	11.33
Epoxy + Al	Hemp	0/90°	7	250 g/m^2^	11	9.16
Epoxy + Al	Glass	0/90°	7	300 g/m^2^	11	10.65

**Table 2:** Technical Properties of the Matrix Material.

Property	BASAT-A 102520	BASAT-B 102520	Mix
Aspect	Greyish thick paste to solid	Clear liquid	Greyish tick paste
Viscosity 25 °C		46–60 mPa·s	10.000 mPa·s
Density at 25 °C	1.5–2.1 g/cm^3^	0.8–1.0 g/cm^3^	1.45–2.0 g/cm^3^

**Table 3:** Cutting parameters used in the experiments.

Spindle speed (rev/min)	1500			3000		
Feed rate (mm/min)	50	75	100	50	75	100

## Data Availability

The original contributions presented in this study are included in the article Further inquiries can be directed to the corresponding author.
